# Assessing Connectivity Between an Overlying Aquifer and a Coal Seam Gas Resource Using Methane Isotopes, Dissolved Organic Carbon and Tritium

**DOI:** 10.1038/srep15996

**Published:** 2015-11-04

**Authors:** Charlotte P. Iverach, Dioni I. Cendón, Stuart I. Hankin, David Lowry, Rebecca E. Fisher, James L. France, Euan G. Nisbet, Andy Baker, Bryce F. J. Kelly

**Affiliations:** 1 Connected Water Initiative Research Centre, UNSW Australia, UNSW Sydney, NSW, 2052, Australia; 2 Australian Nuclear Science and Technology Organisation, New Illawarra Rd, Lucas Heights, NSW, 2234, Australia; 3 Royal Holloway, University of London, Egham Hill, Egham, Surrey TW20 0EX, United Kingdom; 4 School of Environmental Sciences, University of East Anglia, Norwich, Norfolk, NR4 7TJ, United Kingdom

## Abstract

Coal seam gas (CSG) production can have an impact on groundwater quality and quantity in adjacent or overlying aquifers. To assess this impact we need to determine the background groundwater chemistry and to map geological pathways of hydraulic connectivity between aquifers. In south-east Queensland (Qld), Australia, a globally important CSG exploration and production province, we mapped hydraulic connectivity between the Walloon Coal Measures (WCM, the target formation for gas production) and the overlying Condamine River Alluvial Aquifer (CRAA), using groundwater methane (CH_4_) concentration and isotopic composition (δ^13^C-CH_4_), groundwater tritium (^3^H) and dissolved organic carbon (DOC) concentration. A continuous mobile CH_4_ survey adjacent to CSG developments was used to determine the source signature of CH_4_ derived from the WCM. Trends in groundwater δ^13^C-CH_4_ versus CH_4_ concentration, in association with DOC concentration and ^3^H analysis, identify locations where CH_4_ in the groundwater of the CRAA most likely originates from the WCM. The methodology is widely applicable in unconventional gas development regions worldwide for providing an early indicator of geological pathways of hydraulic connectivity.

Unconventional gas production, which is increasingly important to the global energy industry, is the focus of major environmental questions. Debates about the impact of gas production can hinge on claims about CH_4_ leaks and emission, but methodologies to fingerprint gas sources are as yet weak. The production of unconventional gas typically requires the joint extraction of very large quantities of groundwater per day, which can affect groundwater levels in neighbouring aquifers^1^,^2^. Another environmental concern is the impact of gas migration on shallow groundwater resources^1^,^2^,^3^,^4^,^5^. Recent studies around shale gas production sites in the USA report elevated CH_4_ in aquifers up to two kilometres away from the production wells^6^,^7^. More recent studies have argued that this may not be a result of shale gas production^8^,^9^. Although research is largely associated with shale gas developments in the USA^1^,^2^,^3^,^4^,^5^,^6^,^7^,^8^,^9^ similar issues associated with aquifer hydraulic connectivity also accompany CSG developments worldwide. It is clear that a methodology to quantify potential impacts of unconventional gas production needs to be developed, as well as an understanding of existing pathways of hydraulic connectivity, prior to significant gas production.

This research tests the hypothesis that CH_4_ can be used to map zones and identify pathways of hydraulic connectivity between a gas production site and an adjacent freshwater aquifer. Natural pathways of connectivity may be via faults, fracture networks and permeable sedimentary formations^10^. There are also potential pathways of hydraulic connectivity via abandoned exploration wells and faulty well casings^11^,^12^.

Previous studies in the USA near shale gas production have made use of stable isotopes to identify sources of CH_4_ in shallow groundwater^13^,^14^. This study, for the first time, tests the suitability of jointly using δ^13^C-CH_4_, DOC concentration [DOC] and ^3^H activity in the groundwater to assess hydraulic connectivity. These three measurements provide considerable insights into pathways of groundwater and gas movement because:

-  ^  3^ H activities provide information on groundwater residence times and recharge pathways,

 -  [DOC] provides a measure of the carbon inputs, either from the river recharge or the upward  migration of CH_4_ from a coal bed, and

 -   δ^13^C-CH_4_ can be used to characterise the potential sources of the CH_4_ within an aquifer.

The suitability of using ^3^H, [DOC] and δ^13^C-CH_4_ as key parameters for identifying locations where there is hydraulic connectivity between a freshwater aquifer and an underlying unconventional gas reservoir is described in detail below. The methodology is applied to a hydraulic connectivity investigation between the target WCM (on an international scale, a large unconventional gas development) and the overlying CRAA, which supplies water for irrigated agricultural regions, producing a billion dollars worth of commodities. The methodologies presented here are applicable to the many large and geologically similar CSG resources worldwide, including those from the USA, India, China and South Africa^15^, because we measure geochemical parameters that are common to all sedimentary systems.

## Study Area

**Figure 1:**
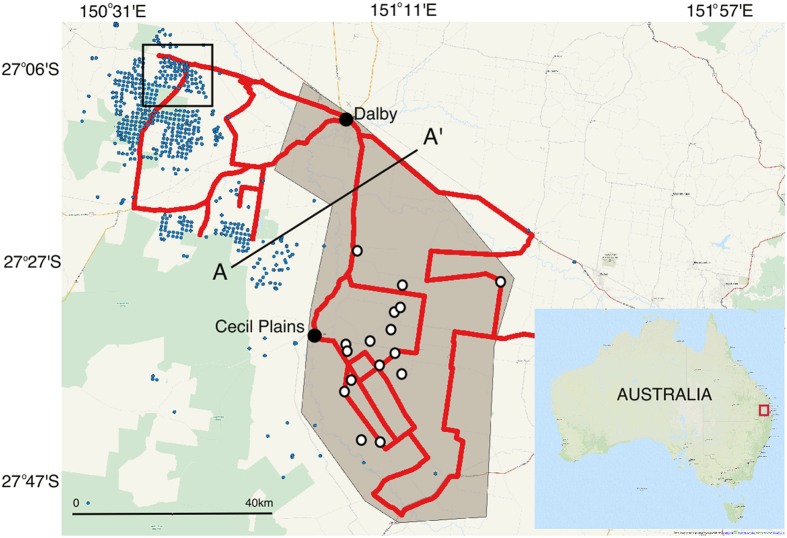
Site map showing the extent of the region investigated within the Condamine Catchment, south-east Queensland. The shaded polygon delineates the extent of the irrigation district^53^. The location of the CSG production and exploration wells are shown as blue^54^ circles and the sampled irrigation bores are shown as white circles. The red line highlights the continuous mobile CH_4_ survey route around the irrigation district. The black square shows the map area in Fig. 3a (Map created in QGIS; data and imagery: MapQuest, Open Street Map and contributors, CC-BY-SA^55^. Modified with Corel Painter 2015^56^).

In Australia, CSG exploration and production have expanded rapidly throughout south-east Qld over the last decade (Fig. 1)^15^. This has generated considerable public concern about the impact on adjacent aquifers used to support irrigated agriculture, stock and domestic water supplies. The proximity of gas extraction to aquifers used for irrigation or domestic water supply is common to many CSG production sites globally. In this study we investigate groundwater from the unconfined CRAA, which has historically supplied 81.4 GL/year of water for irrigated agriculture^16^. This aquifer is located immediately east of the expanding Daandine and Kogan North CSG developments, which began producing in 2006^17^. Currently, within 50 km of the western boundary of the CRAA, there are around 500 producing wells (Fig. 1). The target gas resource for production wells (WCM) starts at approximately 300 m below the ground surface^18^.

There are insufficient baseline CH_4_ data of near-surface ambient air and dissolved gas in groundwater within the Condamine Catchment^19^,^20^,^21^, and our understanding of the extent of hydraulic connectivity between the WCM and the CRAA is limited. Owen *et al.*^20^. used principal component analysis (PCA) to investigate connectivity using historical geochemical major ion data from 879 wells within the CRAA and adjacent Surat Basin. They concluded “in general, no relationships were observed between CSG groundwater in the WCM and the alluvial groundwater.^20^” Major ions migrate between aquifers via advection and there would need to be significant movement of water between formations for geochemical mixing to be apparent. They did not examine [DOC], CH_4_ concentration [CH_4_] or isotopic composition (δ^13^C-CH_4_) within the groundwater. Another study in the region, using ^3^H, dissolved inorganic carbon isotopes (δ^13^C-DIC) and Sr isotopes (^87^Sr/^86^Sr) found that there were limitations in using conventional tracers to assess aquifer connectivity^22^. The authors concluded that “other innovative tracers need to be tested in order to confirm inter-aquifer interactions in CSG environments”^22^.

### Hydrogeological Setting

**Figure 2:**
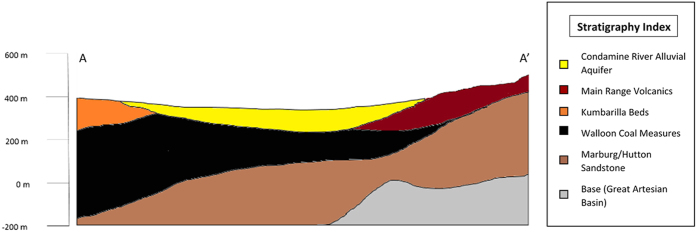
Geological cross section along A-A' in Fig. 1 **(adapted from KCB Final Report**^26^).

The entire study area sits within the Surat Basin, which is part of the Great Artesian Basin (GAB) in south-east Qld (Fig. 1). The units of the GAB, including the WCM, vary locally between semi-confined and confined^23^ and the WCM in places immediately underlie the CRAA (Fig. 2)^24^.

The environment of deposition for the Surat Basin was mainly fluvio-lacustrine during the late Triassic-Jurassic and shallow marine and coastal in the Cretaceous, similar to associated GAB units^25^. The middle Jurassic WCM are a low-rank CSG resource in the Surat Basin^25^. They consist of very fine- to medium-grained sandstone, siltstone, mudstone and coal, with minor calcareous sandstone, impure limestone and ironstone^26^. The coal consists of numerous discontinuous thin lenses separated by sediments of low permeability^27^. The WCM are up to 700 m thick, however the thickness of the coal makes up less than 10% of the total thickness of the unit. The WCM dip gently (~4°) to the west, consistent with the general trend of the Surat Basin in this region. In the region of study, the WCM is thicker (150 m to 350 m) along the western margin of the CRAA, and thins to approximately 50 m in the east, where it outcrops^26^. Also, along the eastern margin of the valley, the CRAA is bounded by the Main Range Volcanics^24^. The WCM overly the Eurombah Formation (a conglomeratic sandstone with minor siltstones and mudstone beds) and underlies the Kumbarilla beds (predominantly sandstone, with lesser mudstone, siltstones and conglomerates)^26^. The unconfined CRAA fills a palaeovalley that was carved through the GAB formations. These reworked eolian and alluvial sediments were deposited throughout the mid-Miocene to the present^23^,^28^. The valley-filling sediments, which have a maximum thickness of 134 m near Dalby^23^,^24^, consist of gravels and fine- to coarse-grained channel sands interbedded with floodplain clays and, on the margins, colluvial deposits.

## Sources of Methane

Within the Condamine Catchment there are many potential sources of CH_4_ that could contribute to the measured [CH_4_] and δ^13^C-CH_4_ in the groundwater and air. These include CH_4_ from:

- the upward migration of gas from the WCM,

- biological activity in the saturated zone beneath rivers and wetlands,

- biological activity in the saturated zone of the CRAA,

- biological activity in the vadose zone (CH_4_ sink),

- biological activity within the borehole, and

- anthropogenic inputs into the atmosphere.

Each of the above CH_4_ sources potentially has a unique δ^13^C-CH_4_ signature^29^. Coal commonly has a portion of the CH_4_ derived from thermogenic origins and therefore contains more ^13^C, resulting in a less negative δ^13^C-CH_4_ signature. In contrast, all the listed biological sources of CH_4_ will be depleted in ^13^C relative to thermogenic sources and atmospheric background, resulting in a more negative δ^13^C-CH_4_ signature^11^,^30^. However, there can be complications in interpreting the isotopes, as various processes can alter the concentration or isotopic composition of the gas. Oxidation of CH_4_ in the vadose zone and atmosphere^31^, groundwater flow fractionation within boreholes^32^, and the mixing of various sources of CH_4_^33^ can alter the isotopic signature. Therefore, improved knowledge of the sources of CH_4_ is required if we are to attribute measured changes in concentration in the groundwater and air to the appropriate sources.

Biological activity in saturated zones produces CH_4_ as a result of methanogenesis. This can occur via either acetate fermentation or reduction of carbon dioxide (CO_2_) (equation (1) and (2) respectively):^31^


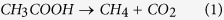



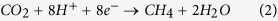


Anaerobic oxidation of CH_4_ (AOM), via reduction of sulphate (SO_4_^2−^) or denitrification of nitrate (NO_3_^−^) may also occur in the saturated zone^34^,^35^,^36^. One of the conditions required for AOM is elevated concentrations of SO_4_^2−^ or NO_3_^−^ in the groundwater. Therefore, testing the groundwater for SO_4_^2−^ or NO_3_^−^ can give an indication as to the potential for AOM to occur. Microbes in the deep aquifer are also responsible for AOM^37^. However, no member of the methanotrophic Euryarchaeota group responsible for anaerobic oxidation has been cultured yet and the various pathways by which AOM occurs are still largely unknown^37^.

Biological activity within the vadose zone yields a net consumption (oxidation) of CH_4_. Oxidation of CH_4_ results in an enrichment of ^13^C, as the methanotrophs preferentially consume ^12^C^31^. This process can go through different reaction intermediates before producing CO_2_ (equation (3)):^31^


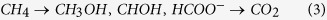


Methanotrophic processes can mask the full extent of the upward migration of CH_4_ because they deplete the concentration of CH_4_ in the subsurface. Oxidation processes in the near surface can also alter the source isotopic signature via fractionation. This allows us to detect methanotrophic activity in a ^12^C rich environment, such as the vadose zone, because the isotopic signature of CH_4_ will be depleted in ^12^C. This is evident in the results presented below.

Within the WCM most of the CH_4_ is adsorbed to the coal, however a small portion can exist in a free state^15^. Methane in its free state is buoyant, and over time may migrate upwards. Since the 1960s groundwater levels within the CRAA have declined^24^ by 25 m and this may have enhanced the upwards migration of CH_4_. The reduction in weight immediately overlying the WCM has the potential to unload the coal interval, releasing a portion of the free state CH_4_. This CH_4_ will have an isotopic signature that is different from near-surface microbiologically produced CH_4_.

## Using Isotopes of Carbon and Hydrogen to Attribute the Source of Methane

To assess the potential of CH_4_ as a marker for hydraulic connectivity, we measured [CH_4_] and δ^13^C-CH_4_ in both the groundwater and air of the Condamine Catchment. The [DOC] was measured in each irrigation borehole to investigate pathways of groundwater flow. Tritium activity was measured in the groundwater to provide insights about groundwater residence times, recharge processes and potential groundwater mixing. Anions (SO_4_^2−^ and NO_3_^−^) were also measured to provide insights into anaerobic processes deep in the aquifer and δ^13^C of dissolved inorganic carbon (δ^13^C-DIC) was measured to give insights into the source of the inorganic carbon in the groundwater.

Atmospheric background concentrations of CH_4_ change with latitude due to changing sources and sinks^38^. The background concentration of CH_4_ at the Cape Grim Baseline Air Pollution Station in Tasmania, Australia (40.683°S), was 1.754 ± 0.002 ppm in 2013^36^. Continuous measurements from the Commonwealth Scientific and Industrial Research Organisation (CSIRO) and Geoscience Australia station at Arcturus, central Queensland (24.027°S) currently range between 1.750 ppm and 1.770 ppm^40^. The isotopic signature value (δ^13^C-CH_4_) commonly reported for ambient background air is −47‰^41^.

Accepted threshold values and isotopic ranges allow us to attribute a source from the measured δ^13^C-CH_4_ obtained for each source of CH_4_ encountered throughout the region^41^. Australian CSG has a δ^13^C-CH_4_ value of less than –60‰ for biologically sourced gas (biogenic CH_4_) and greater than −50‰ for gas sourced from coal at depth (thermogenic CH_4_)^42^. The intermediate zone is classified as being of mixed source. Previous studies have measured the CH_4_ from the WCM using core samples from gas wells. The δ^13^C-CH_4_ readings for these cored samples ranged from −58.5‰ to −45.3‰^25^,^43^,^44^, which indicates that the gas within the WCM is secondary biogenic in origin, with a minor thermogenic component^25^.

## Results and Discussion

### Background ambient air

We established the background [CH_4_] and isotopic signature of air in rural New South Wales (NSW) and Qld by collecting 8 ambient air samples in areas of native vegetation and dryland farming between Narrabri (NSW) and Dalby (Qld). The average background [CH_4_] was 1.774 ± 0.002 ppm and the isotopic signature was −47.0 ± 0.05‰ (n = 8; Supplementary Table S3 online). These values are slightly higher than those reported for the Cape Grim Baseline Air Pollution Station in Tasmania, and at Arcturus in central Qld^39^,^40^.

### Co-produced water storage reservoir

**Figure 3:**
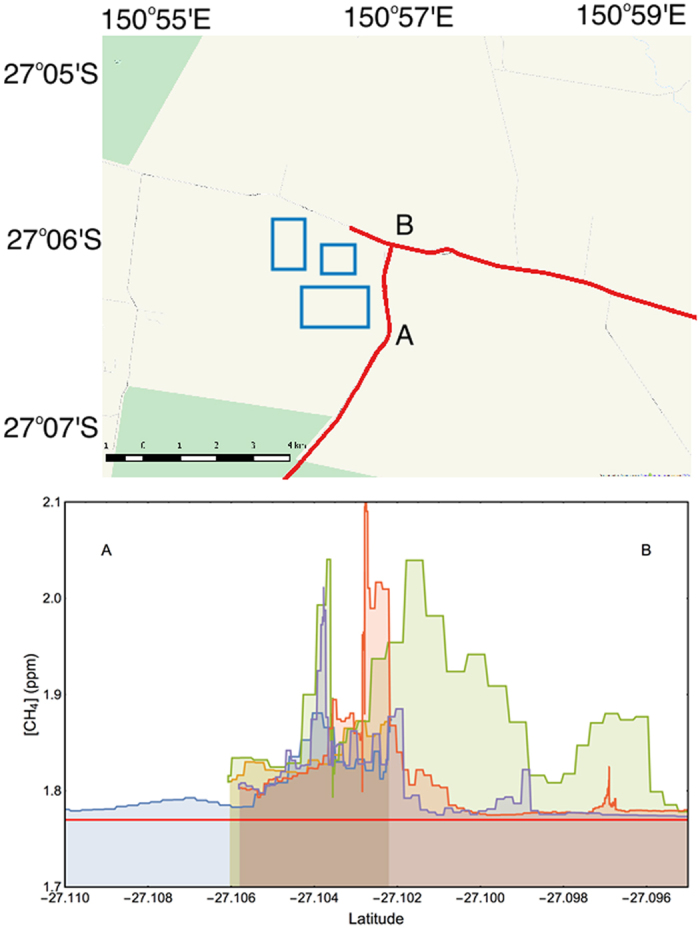
(**a**) A map of CSG developments and co-produced water storage reservoirs. The blue rectangles represent the location of the water storage reservoirs and the red line indicates the route of the mobile survey. The A-B represents the mobile CH_4_ measurement traverse lines, where Tedlar bag samples were collected downwind of the co-produced water reservoir to establish the isotopic composition of CH_4_ extracted from the WCM (Map created in QGIS; data and imagery: MapQuest, Open Street Map and contributors, CC-BY-SA^55^. Modified with Corel Painter 2015^56^). (**b**) CH_4_ concentration measured along the line A-B in the plume downwind of the co-produced water storage reservoir. The horizontal red line indicates the background CH_4_ concentration of 1.774 ppm.

We were unable to measure [CH_4_] directly from CSG well-heads; however, at each production well large quantities of water are co-produced. This water is stripped of most of the CH_4_ and then stored in large holding reservoirs. We were able to indirectly measure the gas from the WCM by analysing the emissions from one such reservoir with dimensions 800 m by 500 m. To determine the signature of the gas being produced from the WCM we surveyed immediately adjacent to and downwind (east) of the reservoir (Fig. 3a). At the closest point the measurements were within 50 m of the reservoir. We detected an area of elevated [CH_4_] that had a peak of 2.107 ppm and a width of 2.3 km.

The [CH_4_] measured during the near-reservoir traverses are plotted in Fig. 3b. Each coloured curtain represents a single survey run in the vehicle. For each run, there are slight differences in the recorded [CH_4_] at the same location. We attribute this to the varied mixing processes associated with the fluctuating winds (both direction and speed).

The isotopic composition of a mixed air sample, which consists of a point source (δ^13^C-CH_4 (s)_; [CH_4 (s)_]) added to background air (δ^13^C-CH_4 (b)_; [CH_4 (b)_]), is determined from:^45^,^46^,^47^


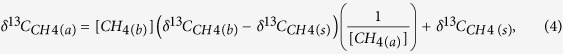


where δ^13^C-CH_4 (a)_ and [CH_4 (a)_] are the isotopic value and concentration measured in the ambient air sample, respectively.

**Figure 4:**
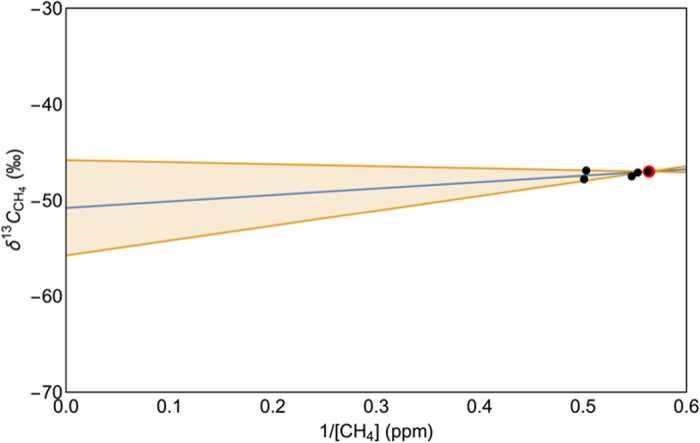
Co-produced water reservoir downwind plume mixing plot. Background air is shown as a red dot, and the downwind plume samples are the black dots. The blue line is the line of best fit for the mixing model (equation (4)) and the 90% confidence interval bands are shown in orange. Source intercept determined for the mixing model is −50.8‰ (90% CI, −55.7‰ to −45.8‰).

For δ^13^C-CH_4 (b)_ and [CH_4 (b)_] we used the average of the air samples collected in areas of natural vegetation for the background values (δ^13^C-CH_4 (b)_ = −47.0‰; [CH_4 (b)_] = 1.774 ppm). The isotopic signature of the off-gassing co-produced water reservoir was then determined by fitting equation (4) to the data using the nonlinear model fitting function in Mathematica^48^ (Fig. 4; Supplementary Table S6 online). To determine the source δ^13^C-CH_4_ value for the WCM three air samples collected along line A-B (Fig. 3), when the wind was blowing from the west directly across the reservoir, were combined with two other samples downwind of the CSG region. Figure 4 shows the mixing model (equation (4)) line of best fit for the combined samples. This yielded a value of −50.8‰ (90% CI, −55.7‰ to −45.8‰) for δ^13^C-CH_4 (s)_. The isotopic signature of the CH_4_ off-gassing from the reservoir indicates that the gas has mixed thermogenic/biogenic origin. The broad confidence interval (−55.7‰ to −45.8‰) is due to a combination of the sample size and measurement error. These 90% confidence interval bounds sit within the δ^13^C-CH_4_ range reported for the WCM^43^. We therefore use the isotopic signature of −50.8‰ as our reference value for the WCM. In the discussion below we use this isotopic signature for the WCM as part of attributing the source of CH_4_ within the aquifers.

### The irrigation district

The irrigation district, to the south of Dalby and the east of Cecil Plains, has a zone of CH_4_ production along the Condamine River, with [CH_4_] peaks as high as 1.930 ppm. However, most of the region is undergoing methanotrophic processes, resulting in a slight decrease in [CH_4_] in the irrigation district. In and around the irrigation district, the continuous CH_4_ survey (Fig. 1) recorded low [CH_4_] values, with the lowest concentration measured being 1.764 ppm. The average [CH_4_] recorded in the continuous survey throughout the district was 1.771 ± 0.007 ppm (n = 8954), slightly lower than the regional atmospheric background (δ^13^C-CH_4 (b) = _−47.0‰; [CH_4 (b)_] = 1.774 ppm).

We were able to further examine the processes occurring in the unsaturated and saturated zones of the aquifer by analysing the [CH_4_] and isotopic signatures within irrigation boreholes in the region.

### Irrigation boreholes

The second data set focused on the groundwater and gas within private irrigation boreholes, details of which are provided in Supplementary Table S4 online.

**Figure 5:**
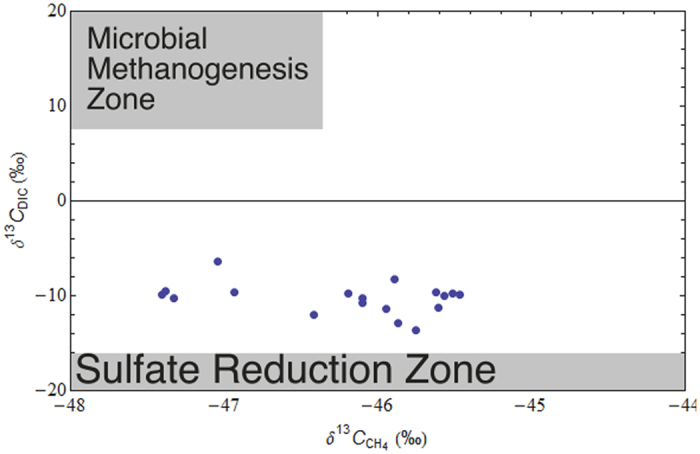
δ^13^C-DIC against δ^13^C-CH_4_ showing no correlation (R^2^ = 0.04).

Our δ^13^C-DIC and δ^13^C-CH_4_ measurements from all irrigation boreholes display no correlation (R^2^ = 0.04), with the values falling outside of the methanogenesis or sulfate reduction zones^7^ (Fig. 5; Supplementary Table S5 online). The DIC in this groundwater is in part from the dissolution of regolith carbonates formed in the vertosols. These calcareous soils are often distributed close to areas with Ca-rich bedrock, such as limestone and basalts, such as the Main Range Volcanics in the study area^49^. Therefore the lack of a correlation between δ^13^C-DIC and δ^13^C-CH_4_ implies that the CH_4_ is from a different source. In other studies microbial CH_4_ from shallow depths has a strong positive correlation between δ^13^C-DIC and δ^13^C-CH_4_^7^. Results obtained for δ^13^C-DIC are 18‰ to 22‰ lighter (more negative) than values expected for DIC sourced from microbial CH_4_ in shallow groundwater.

In this study we use the absence of ^3^H and [DOC] above the detection limit to provide insights into hydraulic connectivity because this combination implies that, in the old groundwater (>70 years), there is another source of [DOC]. Recorded ^3^H activities in the groundwater from irrigation boreholes were generally higher in the Condamine River corridor, and lower compared to modern rainfall values in the central portion and east of the catchment.

**Figure 6:**
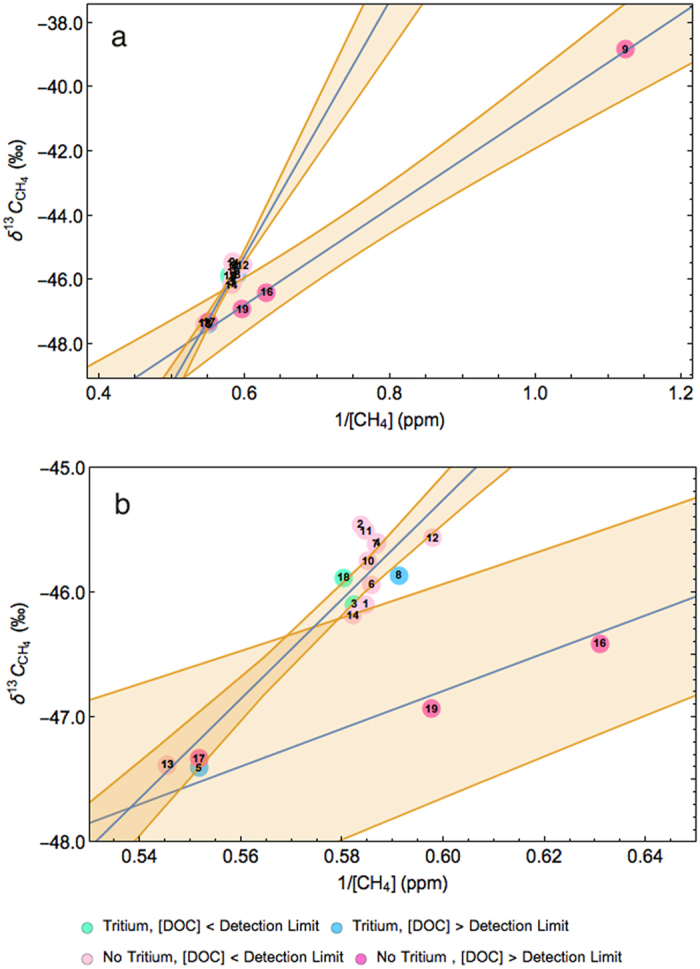
(**a**) A combined mixing plot for the irrigation bores. The regression lines represent either the bores with detectable [DOC] (intercept: −55.9‰ (90% CI, −58.3‰ to −53.4‰)) or the bores with no detectable [DOC] (intercept: −69.1‰ (90% CI, −73.2‰ to −65.0‰)). Tritium activity is indicated by the marker color. All boreholes with no ^3^H activity and detectable [DOC] sit on the regression line with intercept of −55.9‰, indicating gas from the WCM. (**b**) The cluster of bores around 0.55 ppm^−1^ highlights a mixing of parameters at the intercept of the regression lines. This indicates that the absence of ^3^H activity can be used to attribute source but it must be used in tandem with detectable [DOC] values. Mixing model lines of best fit are shown in blue, and the 90% confidence interval bands in orange.

To interpret the CH_4_ measurements from the boreholes we plotted δ^13^C-CH_4_ versus 1/[ CH_4_] (Fig. 6; Supplementary Table S5 online (Fig. 6b highlights the cluster of irrigation borehole samples around 0.55 ppm^−1^ in Fig. 6a.)). Two distinct distributions of points are shown in Fig. 6. These relate to the measured activity of ^3^H, [DOC] > 0.1 mg/L, [CH_4_] and δ^13^C-CH_4_. We assigned samples to subsets for fitting equation (4) to the data (allowing the background values to float) based on the presence or absence of ^3^H activity and [DOC]. Only samples 9, 16, 17 and 19 have ^3^H activity below the quantification limit and [DOC] above the detection limit, and these samples were assigned to subset A. The remaining samples have a mixture of ^3^H activities and detectable [DOC] values, and were assigned to subset B.

Under the assumption that there are only two end members, an estimate of their isotopic signatures can be obtained from a least squares regression fit of equation (4). Subset A has a *y*-axis intercept for the regression line of −55.9‰ (90‰ CI, −58.3‰ to −53.4‰). This is the source signature of the CH_4_ in these boreholes. The isotopic value falls within the range reported for gas from the WCM and is also similar to our recorded value for the WCM from the co-produced water reservoir. The other end member indicated from the line of best fit has a value of −40.7‰ (90% CI, −40.75‰ to −40.74‰), which is consistent with mixing from an oxidised source (for example the vadose zone). The absence of ^3^H activity in the subset A samples indicates that recharge from the river corridor takes >70 years to reach these locations. The elevated [DOC] in subset A cannot be attributed to recharge. This is because in the time that it takes for the recharge to reach these boreholes (>70 years), it is reasonable to assume that biological processes would have already consumed the available DOC. Therefore another source is supplying the detected DOC. Upwards migration of CH_4_ from the WCM would be the most likely source.

Subset A boreholes all had elevated concentrations of DO; including the highest three values recorded in our study (Supplementary Table S5 online). Microbial activity would consume this oxygen^50^. We therefore infer that there is minimal biological activity occurring at depth within the aquifer at these locations. In addition, the SO_4_^2−^ and NO_3_^−^ concentrations in the groundwater across the irrigation boreholes were too low to support AOM (Supplementary Table S5 online). Anaerobic oxidation requires one of these pathways to occur and therefore it is reasonable to assume that oxidation deep within the aquifer is minimal.

In summary for subset A, the absence of ^3^H activity in irrigation boreholes with detectable [DOC], in association with the isotopic value of the regression line within the range of the WCM, suggests local connectivity between the CRAA and the WCM in these areas. The detected CH_4_ is attributed to the upward migration of gas from the WCM.

Subset B has a *y*-axis intercept for the regression line of −69.1‰ (90% CI, −73.2‰ to −65.0‰), which indicates a biological source. The regression line indicates a second end member with an isotopic signature of −29.3‰ (90% CI, −29.33‰ to −29.32‰), which represents mixing with a highly oxidised background (the vadose zone). All samples located in a cluster at 0.55 ppm^−1^ have [DOC] below the detection limit, with the exception of irrigation borehole 8, despite many of these irrigation boreholes being near the river. However, the ^3^H activity indicates that samples 3, 5, 8, and 18 are all sourcing water less than 70 years old, which indicates that these irrigation boreholes are extracting water influenced by near surface processes. Samples 1, 2, 4, 6, 7, 10, 11, 12, and 14 have no recorded ^3^H activity and the [DOC] is below the detection threshold. This indicates that these samples are accessing water >70 years old and the DOC has already been utilised by biological processes.

We attribute the source signature of these samples to biological activity within and near the irrigation boreholes. The low [DOC] and DO measurements in the presence of biologically produced CH_4_ is possibly due to microbiological activity after recharge consuming both the DOC and DO. This biological activity would have produced CH_4_ as a by-product, and this source of CH_4_ dominates the isotopic signature measured in the groundwater at these locations^50^.

Measuring δ^13^C-CH_4_ and [CH_4_] is not enough to assign source, there is also a need to measure both the ^3^H activity and DOC, which is highlighted by samples 5 and 17. Samples 5 and 17 both had [DOC] above the detection limit, however sample 17 had no recorded ^3^H activity, whereas sample 5 did. Sample 13 had no detectable [DOC] and no ^3^H activity measured in the groundwater. We propose that biological processes have consumed the DOC. Sample 5 groundwater has been affected by near surface biological activity, whilst sample 17 shows evidence of upward migration of CH_4_ from the WCM. The difference in these three samples despite their isotopic similarities highlights the need for this combination of measurements in attributing source. The combined method presented here is more robust than the individual measurements because δ^13^C-CH_4_ is difficult to interpret individually in environments where both thermogenic and biogenic methanogenesis is occurring along with methanotrophy.

### Evidence for aquifer connectivity

At our case study site in the Condamine Catchment, the isotopic value of −55.9‰ from the irrigation boreholes with detectable [DOC] is not as ^13^C enriched in CH_4_ as expected from a classical thermogenic source, due to biological processes occurring *in situ*. However, it falls within the range of the isotopic signature from the WCM and is significantly more enriched in ^13^C than the samples from irrigation boreholes with [DOC] below the detection limit. Irrigation borehole samples with no ^3^H activity and detectable [DOC] all sat on the isotopic regression line that fell within the range of values reported for the WCM (−58.5‰ to −45.3‰)^25^,^43^,^44^. The value of this regression had a 5.1‰ difference from our own isotopic signature from the co-produced water reservoir. Thus the isotopic signature from off-gassing air samples collected from irrigation boreholes 9, 16, 17 and 19 (refer Fig. 6), the plume from the co-produced water reservoir, and the literature reported values for gas extracted from coal core samples all sit within the mixed thermogenic/biologic range. This is a strong indicator that the CH_4_ sampled from irrigation boreholes 9, 16, 17 and 19 is from the underlying WCM.

This research has demonstrated that three conditions need to be present to infer that pathways of geological hydraulic connectivity exist in areas of CSG development. The δ^13^C-CH_4_ versus inverse [CH_4_] data must plot on a mixing plot regression line with a *y*-axis intercept value indicative of thermogenic to mixed thermogenic/biogenic gas. However, at high concentrations (low 1/[CH_4_]) it is difficult to assign samples to a specific source-determining regression line. Detectable [DOC] can indicate either river corridor recharge or gas migration upwards from depth. Further information about the probable origin of the CH_4_ and DOC is provided by the ^3^H activity. Detectable [DOC] along with ^3^H activity above the quantification limit indicates relatively young water and near-surface biologically sourced CH_4_. Conversely, [DOC] above the detection limit and ^3^H activity below the quantification limit strongly suggests CH_4_ dominated by the upward migration of gas from the WCM. Our results show that measurements of the isotopic composition of CH_4_, [DOC] and ^3^H activity in the groundwater and CH_4_ in the air can be used as an initial assessment of pathways of geological hydraulic connectivity where an alluvial aquifer overlies coal measures targeted for CSG production.

## Methods

From 20 January 2014 to 1 February 2014 we collected CH_4_ samples from 19 irrigation boreholes. The irrigation boreholes had a gas outlet, which allowed us to pump the gas directly into 3 L Tedlar bags using an SKC 222-2301 battery-operated air pump. This gas was being stripped from the groundwater within the borehole and is representative of the gas within the groundwater. The groundwater was extracted from 35.4 m to 199.9 m within the aquifer. The depth to the water table ranged from 5 to 20 m^51^. No purging of these boreholes was required, as the pumps had been running for 2–3 months.

We also sampled groundwater from all 19 irrigation boreholes in the same period. A sampling tube was installed 2 m inside the pump outlet to minimise air/water interactions. Physio-chemical parameters (pH, Eh, temperature, electrical conductivity and dissolved oxygen (DO)) were checked for stability prior to sampling, with all samples fully stable within minutes.

Sample collection involved an in-line, 0.45 μm, high-volume filter, which was connected to the pump outlet. Samples for δ^13^C-DIC and [DOC] were further filtered through a 0.22 μm membrane and collected in 12 mL glass vials (Exetainers) and 60 mL high-density polyethylene (HDPE) bottles, respectively. Samples for δ^13^C-DIC were refrigerated at 4 °C and [DOC] samples were frozen within 12 hours of collection until they were analysed. Samples for ^3^H analysis were collected in 2 L HDPE bottles. Samples for the SO_4_^2−^and NO_3_^−^ analyses were collected in 125 mL HDPE bottles with no further treatment.

A mobile CH_4_ survey was conducted from 12 March 2014 to 18 March 2014 (Fig. 1). The route covered the irrigation district and CSG developments around Dalby and Cecil Plains to the south, and the location of the irrigation boreholes sampled in January. We sampled the air through Teflon tubing connected to an inlet mounted on top of the vehicle (2.2 m above ground level). This air was passed directly into a Picarro G2301 Cavity Ring-Down Spectrometer (CRDS) (Picarro, Inc., Santa Clara, CA), which measured the [CH_4_] every 5 seconds as we drove along the road at 40 km/h. The precision of the Picarro G2301 is <0.001 ppm over 5 seconds. Simultaneously, GPS location was measured using a Hemisphere GPS (model A21), whilst wind speed and direction data were measured with an S2 Climatronics 2D sonic anemometer, which was also attached to the top of the vehicle. The setup is a modification of methods previously used to determine gas leakage around Boston, USA^52^.

Where we discovered an interval of elevated [CH_4_] individual samples of ambient air were pumped into 3 L Tedlar bags using a KNF NMP 830 KNDC B Micro Diaphragm Gas Pump. The vehicle was stationary and the engine switched off when we pumped air into the Tedlar bags for later analysis in the laboratory (see Supplementary Information online).

## Additional Information

**How to cite this article**: Iverach, C. P. *et al.* Assessing Connectivity Between an Overlying Aquifer and a Coal Seam Gas Resource Using Methane Isotopes, Dissolved Organic Carbon and Tritium. *Sci. Rep.* **5**, 15996; doi: 10.1038/srep15996 (2015).

## Supplementary Material

Supplementary Information
