# Supplementary material for: Assessing Connectivity Between an Overlying Aquifer and a Coal Seam Gas Resource Using Methane Isotopes, Dissolved Organic Carbon and Tritium
Source: Sci Rep. 2015 Nov 4;5:15996. doi: 10.1038/srep15996 (PMC4632156; doi:10.1038/srep15996)
Supplement: Supplementary Information [file srep15996-s1.pdf]

Supplementary Information

Assessing Connectivity Between an Overlying Aquifer and a Coal Seam Gas Resource Using Methane Isotopes, Dissolved Organic Carbon and Tritium

Charlotte P. Iverach, Dioni I. Cendón, Stuart I. Hankin, Dave Lowry, Rebecca E. Fisher, James L. France, Euan G. Nisbet, Andy Baker, Bryce F.J. Kelly.

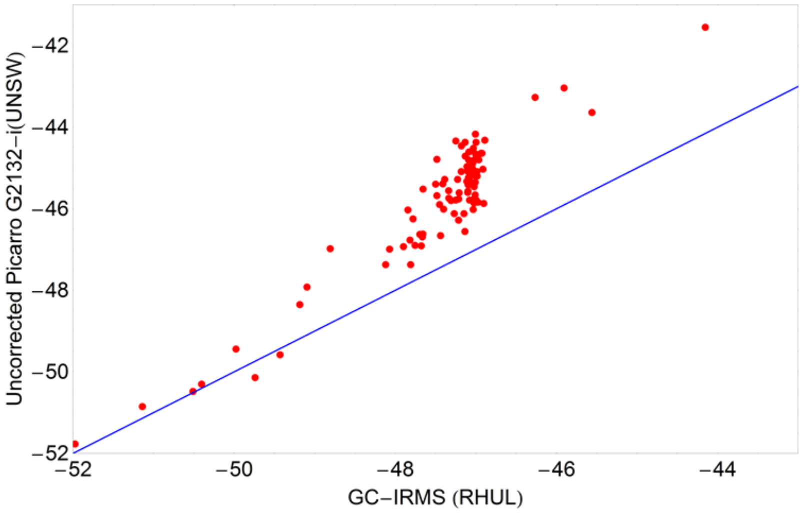

Supplementary Figure S1. Cross-plot of the isotopic values measured using the GC-IRMS against the uncorrected values measured using the G2132-*i*.

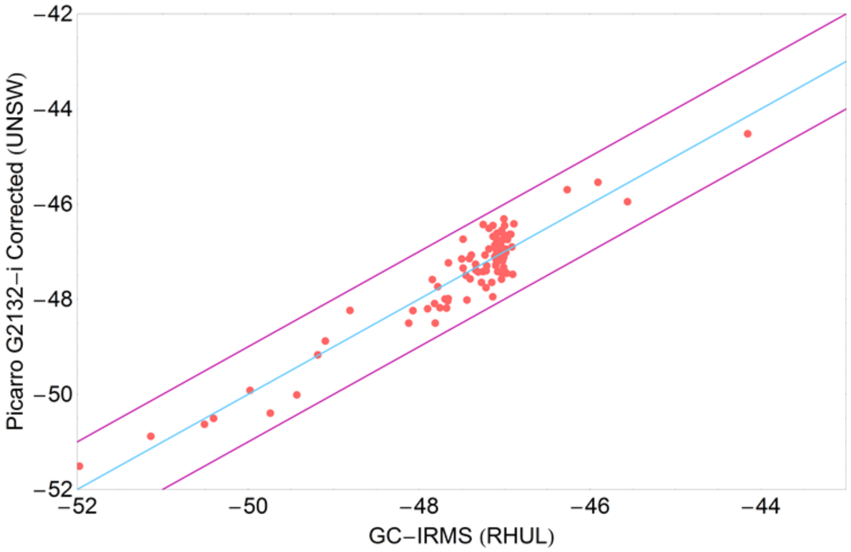

Supplementary Figure S2. A cross-plot of the isotopic values measured using the GC-IRMS and the corrected values from the Picarro G2132-*i* showing that all our data falls within the drift range quoted by the manufacturer.

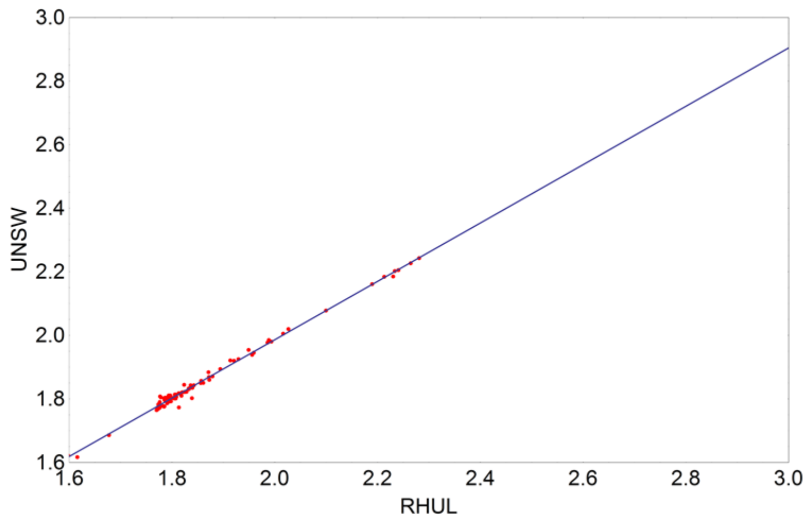

Supplementary Figure S3. A cross-plot of the [CH_4] values measured using the Picarro G1301 and the corrected values from the Picarro G2132-*i* showing a drift of 0.002 ppm. This drift sits within the range of 0.005 ppm quoted by the manufacturer.

Supplementary Table S1. Calibration data for the Picarro G2301.

Australian Campaign Instrument Calibration											
	Known CO_2	1 SD	Meas CO_2	1 SD	Offset (ppm)	Known CH_4	1 SD	Meas CH_4	1SD	Offset (ppb)	H_2O (%)
**Pre-shipping Calibration - 12/2/14**											
**TA-RHUL-2012-Lo1**	389.7	0.05	389.7	0.02	0.00	1834.5	0.2	1833.4	0.2	-1.1	0.009
**TA-RHUL-2012-Med1**	403.5	0.05	403.5	0.02	-0.06	1947.2	0.3	1946.2	0.2	-1.0	0.009
**TA-RHUL-2012-Hi1**	417.9	0.06	417.7	0.02	-0.14	2040.2	0.3	2039.2	0.2	-1.0	0.004
**During Campaign Calibration - Dalby, 13/3/14**											
**CSIRO FB03727**	415.2		415.0	0.02	-0.28	1872.7		1868.9	0.4	-3.8	0.045
**After Campaign - Sydney, 19/3/14**											
**CSIRO FB03727**	415.2		415.2	0.02	-0.08	1872.7		1870.8	0.2	-1.9	0.004
**Back at RHUL Calibration - 26/3/14**											
**TA-RHUL-2011-1**	380.3	0.05	380.4	0.02	0.06	1809.2	0.2	1808.4	0.2	-0.8	0.010
**TA-RHUL-2011-2**	394.9	0.06	395.0	0.02	0.01	1912.4	0.2	1912.4	0.2	0.0	0.013
**TA-RHUL-2011-4**	420.1	0.05	420.0	0.06	-0.10	2091.8	0.2	2092.4	0.3	0.6	0.017

Supplementary Table S2. Data used in establishing the calibration correction for the Picarro G2132-*i*.

Sample	$\delta^{13}C_{CH4}$	error $\delta^{13}C_{CH4}$	$[CH_4]$ (ppm)	UNSW $\delta^{13}C_{CH4}$	RHUL $\delta^{13}C_{CH4}$	RHUL $[CH_4]$	$\delta^{13}C_{CH4}$ diff	$[CH_4]$ diff
ACA1	-45.1	0.05	1.845	-45.1	-47.2	1.824	-2.09	-0.021
ACA3	-44.8	0.05	1.806	-44.8	-47.1	1.805	-2.29	-0.001
ACA6	-44.4	0.05	1.776	-44.4	-47.1	1.785	-2.77	0.009
ACA7	-46.0	0.06	1.851	-46.0	-47.8	1.861	-1.81	0.010
ACA11	-47.9	0.04	2.185	-47.9	-49.1	2.231	-1.18	0.046
ACA12	-47.0	0.04	1.939	-47.0	-48.8	1.956	-1.83	0.017
ACA14	-45.3	0.05	1.792	-45.3	-47.1	1.798	-1.78	0.006
ACA16	-44.3	0.04	1.802	-44.3	-47.3	1.789	-2.91	-0.013
ACA20	-44.5	0.05	1.777	-44.5	-47.2	1.785	-2.72	0.008
ACA22	-45.4	0.07	1.822	-45.4	-47.4	1.828	-2.02	0.006
ACB1	-45.0	0.04	1.783	-45.0	-47.1	1.773	-2.06	-0.010
ACB3	-45.1	0.04	1.774	-45.1	-47.0	1.772	-1.96	-0.002
ACB5	-44.5	0.03	1.765	-44.5	-47.0	1.770	-2.51	0.005
ACB7	-45.1	NA	1.771	-45.1	-47.1	1.775	-1.96	0.004
ACB9	-45.2	0.05	1.767	-45.2	-47.1	1.772	-1.85	0.005
ACB11	-44.2	0.08	1.772	-44.2	-47.0	1.776	-2.84	0.004
ACB13	-44.6	0.07	1.771	-44.6	-46.9	1.775	-2.29	0.004
ACB15	-44.4	0.07	1.775	-44.4	-47.0	1.776	-2.63	0.001
ACC1	-44.6	0.06	1.810	-44.6	-47.1	1.819	-2.48	0.009
ACC3	-45.4	0.09	1.808	-45.4	-47.0	1.777	-1.66	-0.031
ACC4	-45.6	0.05	1.884	-45.6	-47.3	1.871	-1.78	-0.013
ACC5	-44.6	0.04	1.770	-44.6	-46.9	1.771	-2.30	0.001
ACC7	-44.8	0.04	1.782	-44.8	-47.0	1.776	-2.21	-0.006
ACC9	-44.9	0.05	1.786	-44.9	-47.0	1.775	-2.16	-0.011
ACCa 1	-45.2	0.05	1.783	-45.2	-47.0	1.774	-1.78	-0.009
ACCa 2	-45.0	0.07	1.775	-45.0	-47.1	1.775	-2.06	0.000
ACCa 3	-44.8	0.05	1.773	-44.8	-47.0	1.774	-2.17	0.001
ACCa 4	-41.6	0.04	1.520	-41.6	-44.2	1.478	-2.60	-0.042
ACD1	-47.4	0.06	1.945	-47.4	-48.1	1.959	-0.75	0.014
ACD5	-45.8	0.03	1.777	-45.8	-47.0	1.783	-1.13	0.006
ACD6	-46.0	0.05	1.804	-46.0	-47.0	1.786	-1.01	-0.018
ACD15	-46.7	0.05	2.020	-46.7	-47.7	2.027	-0.97	0.007
ACD19	-46.6	0.05	1.804	-46.6	-47.1	1.789	-0.58	-0.015
ACE1	-45.6	0.06	1.803	-45.6	-47.2	1.796	-1.60	-0.007
ACE3	-45.8	0.04	1.808	-45.8	-47.1	1.795	-1.29	-0.013
ACE5	-47.4	0.06	1.925	-47.4	-47.8	1.929	-0.44	0.004
ACE7	-45.8	0.05	1.790	-45.8	-47.0	1.776	-1.21	-0.014
ACE11	-45.9	0.05	1.985	-45.9	-46.9	1.988	-1.03	0.003
ACE13	-46.3	0.06	1.840	-46.3	-47.2	1.838	-0.94	-0.002
ACE14	-46.9	0.06	1.860	-46.9	-47.7	1.873	-0.77	0.013
ACE16	-46.8	0.06	1.894	-46.8	-47.8	1.894	-1.05	0.000
ACE17	-46.9	0.05	1.954	-46.9	-47.8	1.949	-0.85	-0.005
ACE18	-46.9	0.05	1.921	-46.9	-47.9	1.914	-0.97	-0.007
ACE19	-46.6	0.06	2.005	-46.6	-47.7	2.016	-1.04	0.011
ACE20	-45.0	0.04	1.817	-45.0	-47.0	1.815	-2.05	-0.002
ACE21	-45.0	0.06	1.805	-45.0	-46.9	1.779	-1.88	-0.026
ACF2	-45.8	0.04	1.835	-45.8	-47.2	1.840	-1.45	0.005
ACF4	-49.6	0.05	2.202	-49.6	-49.4	2.233	0.15	0.031
ACF6	-45.9	0.06	1.869	-45.9	-47.4	1.872	-1.55	0.003
ACF7	-46.3	0.06	1.920	-46.3	-47.8	1.920	-1.53	0.000
ACF8	-46.1	0.05	1.821	-46.1	-47.1	1.820	-1.03	-0.001
ACF10	-45.8	0.06	1.818	-45.8	-47.3	1.813	-1.46	-0.005
ACF11	-46.7	0.05	1.857	-46.7	-47.4	1.857	-0.78	0.000
ACF12	-47.0	0.05	1.871	-47.0	-48.1	1.879	-1.08	0.008
ACF13	-46.6	0.05	1.977	-46.6	-47.7	1.986	-1.07	0.009
ACF14	-45.4	0.05	1.803	-45.4	-47.0	1.802	-1.61	-0.001
ACF15	-45.6	0.05	1.831	-45.6	-47.1	1.833	-1.50	0.002
ACF16	-46.0	0.06	1.802	-46.0	-47.4	1.839	-1.39	0.037
ACF17	-45.4	0.06	1.801	-45.4	-47.0	1.792	-1.65	-0.009
ACF18	-45.4	0.04	1.773	-45.4	-47.5	1.813	-2.10	0.040
ACG2	-44.8	0.05	1.843	-44.8	-47.5	1.843	-2.70	0.000
ACG4	-44.7	0.04	1.801	-44.7	-47.1	1.807	-2.42	0.006
ACG5	-45.3	0.05	1.811	-45.3	-47.1	1.797	-1.78	-0.014
ACG7	-45.2	0.06	1.791	-45.2	-47.0	1.794	-1.84	0.003
ACG9	-45.0	0.04	1.807	-45.0	-47.1	1.809	-2.14	0.002
ACG11	-45.3	0.05	1.823	-45.3	-47.2	1.828	-1.95	0.005
ACG13	-46.1	0.05	1.843	-46.1	-47.3	1.836	-1.15	-0.007
ACG15	-45.3	0.05	1.822	-45.3	-47.4	1.825	-2.11	0.003
ACG16	-51.8	0.07	2.227	-51.8	-52.0	2.264	-0.21	0.037
ACG20	-45.8	0.05	1.819	-45.8	-47.3	1.818	-1.51	-0.001
ACG22	-45.4	0.05	1.811	-45.4	-47.1	1.794	-1.69	-0.017
ACG24	-45.5	0.05	1.796	-45.5	-47.0	1.785	-1.56	-0.011
ACG26	-48.4	0.05	1.980	-48.4	-49.2	1.993	-0.84	0.013
ACG28	-50.9	0.06	2.205	-50.9	-51.1	2.241	-0.29	0.036
ACG32	-50.1	0.05	2.184	-50.1	-49.7	2.213	0.40	0.029
ACG34	-50.3	0.06	2.243	-50.3	-50.4	2.281	-0.10	0.038
ACG36	-49.4	0.06	2.078	-49.4	-50.0	2.100	-0.54	0.022
ACG38	-50.5	0.05	2.161	-50.5	-50.5	2.189	-0.03	0.028
ACG40	-45.5	0.05	1.850	-45.5	-47.7	1.856	-2.14	0.006
ACG42	-45.6	0.04	1.786	-45.6	-47.1	1.790	-1.53	0.004
5	-45.7	0.04	1.833	-45.7	-47.3	1.833	-1.60	0.000
13	-45.8	0.06	1.814	-45.8	-47.0	1.806	-1.26	-0.008
15	-45.7	0.04	1.801	-45.7	-47.5	1.805	-1.80	0.004
16	-44.7	0.05	1.774	-44.7	-47.0	1.776	-2.34	0.002
17	-45.9	0.05	1.802	-45.9	-47.0	1.785	-1.16	-0.017
19	-45.4	0.06	1.777	-45.4	-47.0	1.777	-1.66	0.000

Supplementary Table S3. Samples used to establish regional background conditions.

Sample	$[CH_4]$ (ppm)	$\delta^{13}C_{CH4}$
ACB 1	1.773	-47.1
ACB 3	1.772	-47.0
ACB 5	1.770	-47.0
ACB 7	1.775	-47.1
ACB 9	1.772	-47.1
ACB 11	1.776	-47.0
ACB 13	1.775	-46.9
ACB 15	1.776	-47.0
Average Background	1.774	-47.0

Supplementary Table S4. Borehole details from the QLD Government database.

Sample	Date Installed	Casing	Elevation (asl)	Depth (m)	First slot (m)
1	9-Jun-01	Steel	345.6	64.0	62.5
2	8-Sep-71	Steel	345.9	56.5	56.5
3	14-Sep-57	Steel	No data	199.9	41.0
4	3-Aug-71	Steel	367.3	43.6	38.9
5	18-Mar-84	Steel	369.2	43.3	37.9
6	1-May-74	Steel	360.0	125.5	59.1
7	12-Sep-67	Steel	359.1	35.4	33.7
8	29-Jul-85	Steel	337.6	68.0	55.2
9	17-May-85	Steel	358.4	47.6	40.5
10	15-Feb-84	Steel	327.2	52.1	47.3
11	9-Jul-74	Steel	351.0	75.3	Perforated
12	16-Feb-78	Steel	358.2	85.6	74.5
13	21-Mar-74	Steel	354.5	98.1	Perforated
14	4-Sep-83	Steel	354.4	95.7	91.2
15	5-Dec-81	Steel	361.5	46.3	42.7
16	19-Sep-95	Steel	361.8	61.6	57.9
17	25-Nov-78	Steel	363.4	80.2	67.2
18	20-Nov-88	Steel	360.8	48.4	40.7
19	17-Apr-83	Steel	373.0	36.0	31.9

Supplementary Table S5: Irrigation bore geochemical data as well as [CH_4] and $\delta^{13}C$-CH_4.

Sample	[CH_4] (ppm)	1/[CH_4] (ppm)	$\delta^{13}C_{CH4}$	$\delta^{13}C_{DIC}$	[SO_4^{2-}] (mg/L)	[NO_3^-] (mg/L)	DO (mg/L)	[DOC] (mg/L)	^3H (TU)
1	1.710	0.585	-46.1	-10.3	34.0	1.60	1.94	< 0.1	0.02
2	1.713	0.584	-45.5	-9.9	32.0	1.40	1.87	< 0.1	0.01
3	1.717	0.583	-46.1	-10.8	37.0	1.30	2.00	< 0.1	0.11
4	1.703	0.587	-45.6	-11.2	3.3	1.40	5.02	< 0.1	0.03
5	1.813	0.552	-47.4	-9.9	3.2	2.30	6.50	> 0.1	0.16
6	1.707	0.586	-45.9	-11.4	14.0	1.20	5.51	< 0.1	0.01
7	1.705	0.587	-45.6	-9.6	8.2	1.40	4.85	< 0.1	0.03
8	1.691	0.591	-45.9	-12.9	47.0	1.50	4.64	> 0.1	0.19
9	0.889	1.125	-38.8	-10.1	11.0	1.20	7.61	> 0.1	0.04
10	1.709	0.585	-45.8	-13.6	29.0	2.10	0.91	< 0.1	0.03
11	1.710	0.585	-45.5	-9.8	34.0	1.50	3.10	< 0.1	0.01
12	1.673	0.598	-45.6	-10.0	14.0	1.30	6.45	< 0.1	0.01
13	1.833	0.545	-47.4	-9.5	22.0	1.30	2.57	< 0.1	0.04
14	1.718	0.582	-46.2	-9.8	32.0	1.40	4.64	< 0.1	0.04
15	1.807	0.553	-47.0	-6.4	5.0	0.05	8.20	Unacceptable uncertainty	Unacceptable uncertainty
16	1.585	0.631	-46.4	-12.0	7.3	1.20	8.53	> 0.1	0.02
17	1.813	0.552	-47.3	-10.2	8.0	1.30	4.31	> 0.1	0.01
18	1.723	0.580	-45.9	-8.3	6.9	1.40	5.37	< 0.1	0.1
19	1.673	0.598	-46.9	-9.6	55.0	2.40	6.39	> 0.1	0.01

Supplementary Table S6: $\delta^{13}C$-CH_4 and [CH_4] data used for the mixing plot in Figure 4.

Sample	[CH_4] (ppm)	1/[CH_4] (ppm)	$\delta^{13}C_{CH4}$
ACE7	1.776	0.563	-47.0
ACE8	1.807	0.553	-47.1
ACE10	1.830	0.547	-47.5
ACE11	1.988	0.503	-46.9
ACE12	1.996	0.501	-47.8

Data Analysis and Quality Control.

The mobile Picarro G2301 was calibrated before, during and after the field survey using 6 different laboratory primary standards at RHUL (Table S1). All of these had an offset that fell within the precision range of the instrument as specified by the manufacturer. Two calibrations during the survey were done using a Southern Ocean Air standard FB03727 provided by CSIRO. The offset was larger with these calibrations, possibly due to the effect of out-of-lab conditions.

The air samples from January and March (that were stored in Tedlar bags) were analysed at UNSW, Sydney, Australia and Royal Holloway, University of London (RHUL), UK. At RHUL, [CH_4] was measured using a Picarro G1301 CRDS, which is calibrated to the National Oceanic and Atmospheric Administration (NOAA) NOAA04 scale for CH_4, using reference gases calibrated by the NOAA Earth System Research Laboratory (ESRL).[57] The precision of these measurements was taken as one standard deviation of the target gas measurements made twice a week and was 0.00035 ppm. Each sample was analysed for 4 minutes on the Picarro G1301 and the concentration value was taken as the mean of the last 2 minutes 20 seconds of the measurement. Analysis of $\delta^{13}C$-CH_4 was carried out using a modified gas chromatography isotope ratio mass spectrometry (GC-IRMS) system (Trace Gas and Isoprime mass spectrometer, Isoprime Ltd.) with 0.05‰ repeatability.[58] All measurements were made in triplicate.

At UNSW, air samples (stored in Tedlar bags) were analysed for [CH_4], $\delta^{13}C$-CH_4, [CO_2] and water (H_2O) concentration on a Picarro G2132-*i* isotope analyser. The precision of this instrument for $\delta^{13}C$-CH_4 > 1.8 ppm is < 0.8‰ over 5 minutes, and < 0.5‰ over 15 minutes. The maximum drift expected in the $\delta^{13}C$-CH_4 value over 24 hours is < 2‰ at 1.8 ppm. The isotopic data presented here are expressed in δ notation (‰) on the international scale, V-PDB (Vienna Pee Dee Belemnite). It is defined as (Supplementary equation (S1)): [57]

$$\delta^{13}C\text{-}CH_4 = [(R_{sample}/R_{standard}) - 1] \times 1000 \quad \text{(S1)}$$

The precision in $[CH_4]$ is 0.005 ppm + 0.05 % of the measured ^{12}C and 0.001 ppm + 0.05 % of the measured ^{13}C.

The ambient air samples from the January and March field campaigns were analysed in March. The air was passed into the Picarro G2132-*i* via a Teflon tube with a simple open/close valve system that allowed either outside air or the sample in the Tedlar bag. Each sample was analysed for 10 minutes, which allowed for stabilization of the readings. Values were taken as the mean over a 2-minute period once the readings were stable. In between each sample, ambient air was run through the Picarro for at least 5 minutes so as to leave clear markers between each bag sample and to avoid contamination from previous bags.

To calibrate the Picarro G2132-*i* we measured the 94 Tedlar bag samples on both the G2132-*i* and the GC-IRMS system at RHUL.[58] A cross-plot of the isotopic values obtained on the GC-IRMS versus the Picarro G2132-*i* is shown in Figure S1. The 1:1 line is shown in the graph. At lower $\delta^{13}C\text{-}CH_4$ values the Picarro G2132-*i* readings match the GC-IRMS readings. However, as the isotopic values became more enriched in ^{13}C the Picarro G2132-*i* readings drifted upwards away from the 1:1 line. We performed a linear correction on the isotopic values obtained by the Picarro G2132-*i* and found that this drift was within ±1‰ bounds of the corrected 1:1 line, resulting in a total drift of 2‰ (Figure S2). This drift of 2‰ on the Picarro G2132-*i* can be clearly seen when there were numerous samples run with very similar $\delta^{13}C\text{-}CH_4$ values. In Figure S1 it is clear that when the isotopic value on the GC-IRMS is -47‰ the drift over 24 hours on the Picarro G2132-*i* is between -46‰ and -48‰. This falls within the drift of < 2‰ over 24 hours quoted by the manufacturer.

A linear correction was also obtained for the Picarro G2132-*i* $[CH_4]$ against a NOAA standard calibrated Picarro G1301 at RHUL using the 94 ambient air samples (Figure S3). These

values were found to have a total precision of 0.002 ppm. This value falls within the precision quoted in the manufacturer's specifications.

The δ^{13}C-DIC samples were analysed at the Australian Nuclear Science and Technology Organisation (ANSTO), Sydney using a Delta V Advantage mass spectrometer, and a GasBench II peripheral. The results are reported as per mil deviations from International Atomic Energy Agency (IAEA) secondary standards that have been certified relative to V-PDB for carbon with a precision of ± 0.3‰. The [DOC] samples were analysed at UC-Davis Stable Isotope Facility, USA with results corrected based on laboratory standards calibrated against National Institute of Standards and Technology (NIST) Standard Reference Materials. Samples were run using a total organic carbon (TOC) analyser connected to a PDZ Europa 20-20 IRMS using a GD-100 Gas Trap interface. The ^{3}H samples were analysed at ANSTO and are reported as tritium units (TU). The water samples were distilled and enriched electrolytically prior to analysis by liquid scintillation. Tritium activity was measured by counting beta decay in a liquid scintillation counter (LSC) and had a combined standard uncertainty of ± 0.03 TU. Only samples that measured above the quantification limit (> 0.04 TU) were considered to have measurable ^{3}H activity.

(57) Dlugokencky, E.J. *et al.* Conversion of NOAA atmospheric dry air CH_4 mole fraction to a gravimetrically prepared standard scale. *J. Geophys. Res.* **110,** 1-8, DOI: 10.1029/2005JD00635 (2005).

(58) Fisher, R., Lowry, D., Wilkins, O., Sriskantharajah, S. & Nisbet, E.G. High-precision automated stable isotope analysis of atmospheric methane and carbon dioxide using continuous-flow isotope-ratio mass spectrometry. *Rapid Commun. Mass Spectrom.* **20**(2), 200-208, DOI: 10.1002/rcm.2300 (2006).
